# Executive Function and Working Memory Deficits in Females with Fragile X Premutation

**DOI:** 10.3390/life13030813

**Published:** 2023-03-17

**Authors:** Osnat Segal, Tamar Kowal, Yonit Banet-Levi, Lidia V. Gabis

**Affiliations:** 1 Department of Communication Disorders, The Stanley Steyer School of Health Professions, Sackler Faculty of Medicine, Tel-Aviv University, Tel Aviv 6997801, Israel; 2 Sagol School of Neuroscience, Tel-Aviv University, Tel Aviv 6997801, Israel; 3 Maccabi Healthcare, Tel Aviv 6200522, Israel; 4 Keshet Autism Center Maccabi Wolfson, Holon 5822012, Israel; 5 Sackler Faculty of Medicine, Tel-Aviv University, Tel Aviv 6997801, Israel

**Keywords:** fragile X premutation, females, executive functions, phonological working memory, clinical genetics, adults

## Abstract

The Fragile X premutation is a genetic instability of the *FMR1* gene caused by 55–199 recurrences of the CGG sequence, whereas there are only 7–54 repeats of the CGG sequence in the normal condition. While males with the premutation of Fragile X were found to have difficulties in executive functions and working memory, little data have been collected on females. This study is among the first to address executive functions and phonological memory in females with the Fragile X premutation. Twenty-three female carriers aged 20–55 years and twelve non carrier females matched in age and levels of education (in years) participated in this study. Executive functions and phonological memory were assessed using the self-report questionnaire The Behavior Rating Inventory of Executive Function (BRIEF) and behavioral measures (nonword repetitions, forward and backward digit span). Females who were carriers of the premutation of the FMR1 gene reported less efficient executive functions in the BRIEF questionnaire compared to the control group. In addition, a relationship was found between the number of repetitions on the CGG sequence of nucleotides, nonword repetitions, and forward digit span. The findings suggest that the premutation of Fragile X in females affects their performance of executive functions and may have impact on everyday functioning.

## 1. Introduction

### 1.1. Fragile X

The Fragile X Syndrome (FXS) is the most common cause of intellectual disability and autism as well as additional difficulties. It is caused by the absence of the *FMR1* protein [1,2,3,4,5,6,7,8,9,10]. The syndrome is caused by more than 200 repeats of a sequence of three nucleotides, Cytosine–Guanine–Guanine (CGG), located in the promoter region on the long arm of the X chromosome [9,10,11,12,13,14,15]. The expansion of the CGG trinucleotide repeats leads to methylation and the subsequent silencing of the gene, which leads to loss of expression of the *FMR1* protein. The *FMR1* protein is an RNA binding protein involved in several processes, including neuronal plasticity, and functioning of neuronal networks [9]. When the number of CGG repetitions in the *FMR1* gene is 55–199, the gene is not stable when transmitted between generations, and may expand to full mutation [16,17,18,19]. The prevalence of the premutation is estimated at 1:260–813 in males and 1:110–259 in females [15,16,20,21]. In Israel, the prevalence of the premutation in women is 1:101–264 [21,22,23,24]. Some researchers refer to people with the premutation as carriers of FXS [17].

In the past, the phenotype of carrier status was considered asymptomatic, and its main significance was the risk of a further increase to a full mutation in the offspring of the carriers [22,25]. In recent studies, however, unique disorders related to the premutation in the *FMR1* gene have been reported, including the Fragile X-associated primary ovarian insufficiency (FXPOI) and Fragile X-associated tremor/ataxia syndrome (FXTAS), with abnormal gait, memory, and executive dysfunction [6,10,11,12,18,25,26]. FXTAS has been reported in 40% of the males and 8%–16% of the females carrying the premutation and who are above 50 years of age [6,18,26,27]. Similar to the full-mutation condition, the phenotype of females with the premutation may differ from that of male carriers because of possible inactivation of the X chromosome with the mutation [26,28,29,30].

The mechanism causing the deficits in premutation differs from the full mutation. In *FMR1* full mutation, the Fragile X Syndrome is caused by the absence of the *FMR1* protein (FMRP) and its functioning. In the premutation, the Fragile X protein (FMRP) levels are normal or slightly reduced, but *FMR1* messenger RNA (mRNA) levels are 2–10-fold higher than the norm [12,13,14,20,31,32], depending on the number of repetitions in the premutation range [1,12,13]. The disorders related to the premutation have been considered to be caused by mRNA toxicity, with higher *FMR1* mRNA, which may cause the death of nerve cells and atrophy of brain regions [6,32,33]. The RNA gain-of-function model proposes that CGG repeats are toxic through the binding and sequestration of specific RNA binding proteins. In contrast, the repeat-associated non-AUG (RAN) translation model proposes that expanded CGG repeats cause pathogenicity through their translation into toxic proteins [2,8,9]. In individuals with the Fragile X premutation, it has been suggested that the local axonal translation (LAN) of mRNA is altered, leading to the accumulation of toxic mRNA species in dendrites and axons. In addition, the *FMR1* premutation allele may also cause neuronal dysfunction by altering the expression of other genes and affecting neurotransmitter signaling pathways. It has been suggested that these processes, among others, contribute to the cognitive difficulties seen in individuals with the Fragile X premutation [2,11].

Several studies have demonstrated that some premutation carriers exhibit mild problems in physical, cognitive, and emotional domains without the symptoms of FXTAS [9,12,17,20]. Premutation carriers reported higher levels of obsessive-compulsive symptoms, depression, and anxiety [9]. Some evidence of dyscalculia was found in female carriers [22]. However, there is a paucity of data on executive functions that may be related to learning disabilities in female carriers of the premutation, and the relationship between executive functions and the size of the mutation (number of CGG repeats).

### 1.2. Executive Functions and Phonological Working Memory in FXS

Executive functions represent cognitive abilities that underlie goal-oriented behaviors including, for example, cognitive flexibility, inhibition, attention shifting, emotional regulation, working memory, and initiation [34,35,36]. Measures of executive functions include self-report questionnaires, initiation and production of verbal information measured through tasks of verbal fluency, mental flexibility and attention measured through the Trail Making Test, and working memory measured assessed with digit span and nonword repetition [20,36]. Working memory and other executive functions are interrelated and work together to achieve a desired goal [37].

One executive function crucial for language learning is phonological working memory [38,39], which is used to temporarily store verbal acoustic information for immediate use. Phonological working memory is often required to process information [36,37,40,41,42,43,44,45,46,47] in order to maintain short-term plans and intentions so that the executive system can use them and organize behavior in a coherent and goal-oriented manner [29,43].

Poor performance on nonword span tasks constitutes a behavioral marker for an existing language impairment and/or a history of language impairment [48,49]. Phonological working memory has been identified as a major component of human intelligence and is affected by various neurogenetic disorders, including schizophrenia, FXS, and autism [1,33].

Some studies have found deficits in executive functions among people with the full mutation. For example, Hooper et al. (2008) reported deficiencies in executive functions such as working memory, inhibition, and cognitive flexibility among boys with FXS (aged 7–13 years) compared to typically developing boys matched for mental age [34]. Whether such deficiencies also exist among premutation carriers is a question that has been the focus of some studies in recent years [12,13,14,15,20,27,50]. Moore et al. (2004) compared male premutation carriers and an IQ- and age-matched control group, and found differences between the groups in executive functions, such as cognitive flexibility and attention [20].

The findings regarding impaired working memory in carriers of the premutation have been inconsistent. Among male carriers, some researchers found greater impairment of phonological working memory [1,14,32] and a positive correlation between the level of impairment and the number of repeats in the *FMR1* gene [1,32]. The higher the number of repeats, the more severe the deficit. The participants had normal intellectual abilities but showed deficits in measures of phonological working memory. Other researchers found no significant difference in working memory abilities between noncarrier males and asymptomatic premutation male carriers [29].

Few studies have examined female carriers. Hunter et al. (2008) found no differences between a group of male and female premutation carriers and a control group under 50 years of age [51]. Hashimoto et al. (2011), however, tested verbal working memory abilities during functional magnetic resonance imaging (fMRI). They found that female and male carriers’ performances were similar to those of the control group, but there were differences in cerebral activation patterns and neuroanatomical abnormalities in areas attributed to working memory function—that is, the right ventral inferior frontal cortex and the left premotor/dorsal inferior frontal cortex. The reduction in activity may be the neurological basis for impairments in memory and other executive functions [6]. Differences in the brain were also found in the structure and function of the cerebellum, which is an area linked to executive functions in carriers compared to a control group [30]. In addition, Yang et al. (2013) found subtle impairments in executive functions, including deficits in working memory among asymptomatic premutation female carriers who were over the age of 50 compared to a control group matched for age and years of education [52].

In sum, difficulties in phonological working memory have been found in males and females with FXS at different ages [13,31] and among premutation carriers with normal cognitive abilities [1,6,14,32,52]. Because this ability is important for many cognitive, verbal, and executive functions involved in everyday life [38,39,40,41,42,44,45] and because difficulties in this ability has been investigated among male premutation carriers but rarely among female premutation carriers [52,53], the present study focused on working memory abilities among females with the Fragile X premutation.

### 1.3. The Present Study

The primary aim of the present work was to test executive functions and phonological working memory among females with the Fragile X premutation. A secondary aim was to examine whether there is a relationship between the number of repeats of the CGG sequence in the *FMR1* gene, executive functions, and phonological working memory. We assumed that (a) females who are carriers of the premutation would exhibit reduced performance in executive functions and working memory compared to the control group without the premutation; and (b) there would be an association between the number of repeats of the CGG sequence in the *FMR1* to the behavioral measures of executive functions and phonological working memory.

## 2. Materials and Methods

### 2.1. Participants

Twenty-three females who were carriers of the Fragile X premutation participated in the present study. None had FXTAS. They had participated in voluntary genetic testing during pregnancy or following a diagnosis of their child with Fragile X (n = 15). The number of repeats of CGG in the *FMR1* gene in this group ranged between 55 and 180 (M = 100.7 repeats, SD = 42.3). Their age range was between 25 and 53 years (M = 39.26 years, SD = 7.49). They had between 12 and 22 years of education (M = 16.2 years of education, SD = 3.21), and all had no known cognitive difficulties according to their self-report. The control group included 12 females with normal *FMR1* alleles, according to test results performed before or during pregnancy. Their age range was between 23 and 49 years (M = 36.5 years, SD = 7.14). They had between 12 and 20 years of education (M = 16.5 years of education, SD = 2.39). A *t*-test for independent samples found no significant differences between the two groups in age [t(33) = 1.051, *p* = 0.301] and years of education [t(33) = (−0.288), *p* = 0.775)]. All females spoke Hebrew as their main language, had normal or corrected to normal vision and no hearing difficulties according to their self-report.

### 2.2. Assessment Tools

#### 2.2.1. Working Memory

Digit Span Test. The digit span test included the digit span forward test and the digit span backward test. The former examined the ability to temporally store auditory information, and the latter involved the ability to manipulate this information by recalling the heard digits in reverse order [54].

FriGvi. The FriGvi battery tests [55] were developed in Hebrew to assess phonological working memory and its effects. In the current study, four subtests were used to assess working memory: (a) basic word span with two-syllable words; (b) a word span for two-syllable words that were phonologically similar, differing from each other only in one phoneme; (c) a word span for four-syllable words; and (d) a word-span test with two-syllable nonwords. Calculating the difference in scores between the basic word-span test and the other subtests provides a measure for assessing the phonological-similarity effect, the length effect, and the lexical effect, respectively [55,56].

#### 2.2.2. Executive Functions

Trail Making Test (TMT). This neuropsychological test is used to screen for neuropsychological deficits. The test assesses visual attention and task switching [57,58] and includes two parts. In the first part of the test, the participant is asked to draw lines connecting 24 numbers in ascending order. In the second part of the test, the participant needs to draw lines connecting 24 numbers and letters in alternating numerical and alphabetical order. The numbers and letters are arranged in semi-random order. The participant is instructed to perform the task as quickly and accurately as possible without lifting her pencil from the page. The measure of performance is the completion time for each part of the test.

The Behavior Rating Inventory of Executive Function—Adult Version (BRIEF-A). This self-report questionnaire evaluates different aspects of executive functions and self-regulation in daily life, namely, inhibitory control, cognitive and behavioral flexibility, emotional regulation, self-monitoring in social contexts, the ability to initiate activity, working memory, planning and organization of cognition and problem solving, organization of materials, and monitoring of problem solving [59]. The participant needs to indicate whether a particular behavior occurs *often*, *sometimes*, or *never*. The Hebrew version of the BRIEF-A was used. The questionnaire was translated into Hebrew and cross-cultural adjustments were made [60].

Verbal Fluency. Verbal fluency tests require fast retrieval of words from memory by given semantic categories (e.g., animals), which is termed *semantic verbal fluency*, or by given phonemic categories (letters), which is termed *phonemic verbal fluency*. This particular test includes include the retrieval of words that start with the Hebrew letters Gimel (/g/), Bet (/b/) and Shin (/S/), and retrieving items belonging to the categories of animals, fruits, vegetables, and vehicles (each category separately). The test was developed in Hebrew and includes norms for Hebrew speakers [61].

### 2.3. Procedure

All females were tested in a quiet room in their homes after signing the consent form. They first filled out a background questionnaire that included questions about speech and language difficulties during childhood, reading acquisition, second language acquisition, and learning difficulties. They were then tested using the tests described above in quasi-random order, alternating between auditory and visual tasks to reduce fatigue.

Detailed instructions and examples were given before each test. The participants were given small breaks of five minutes between tests. The session lasted approximately half an hour. The genetic information of the females in the experiment group was taken from the participants’ medical records with their consent. The genetic information of the control group was from test results that they had performed before or during pregnancy.

This study was approved by the Helsinki committee of the Edmond and Lily Safra Children’s Hospital (9187-11-SMC) and by the ethics committee of Tel Aviv University (6 May 2019).

## 3. Results

The results of the Fragile X premutation group and the control group on the working memory tests (digit span test, FriGvi, TMT) and on the executive function tests (BRIEF-A, verbal fluency) are summarized in Table 1. Supporting information is presented in the section of Supplementary Materials. The following High scores in the BRIEF questionnaire indicate a higher frequency of reports of difficulties related to executive functions. In contrast, for the working memory tests (digit span and FriGvi) and the verbal fluency tests, high scores indicate better abilities in those areas. Faster performance on the TMT indicates better performance on the test.

**Figure 1:**
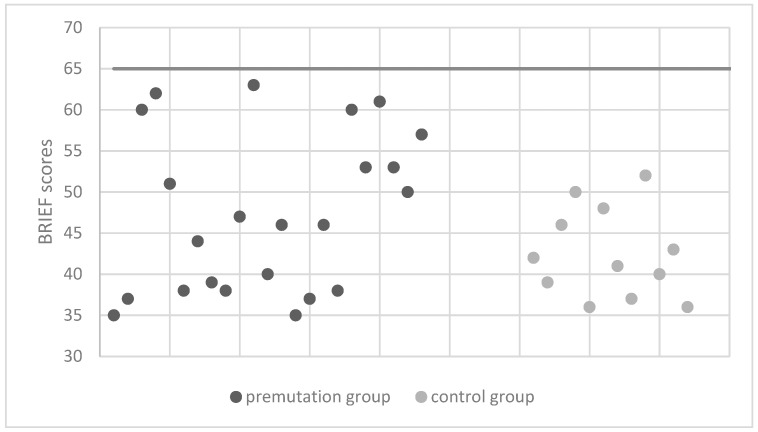
Distribution of standard scores in the BRIEF-A questionnaire for: the premutation group and the control group. The black line represents the norm limit.

**Figure 2:**
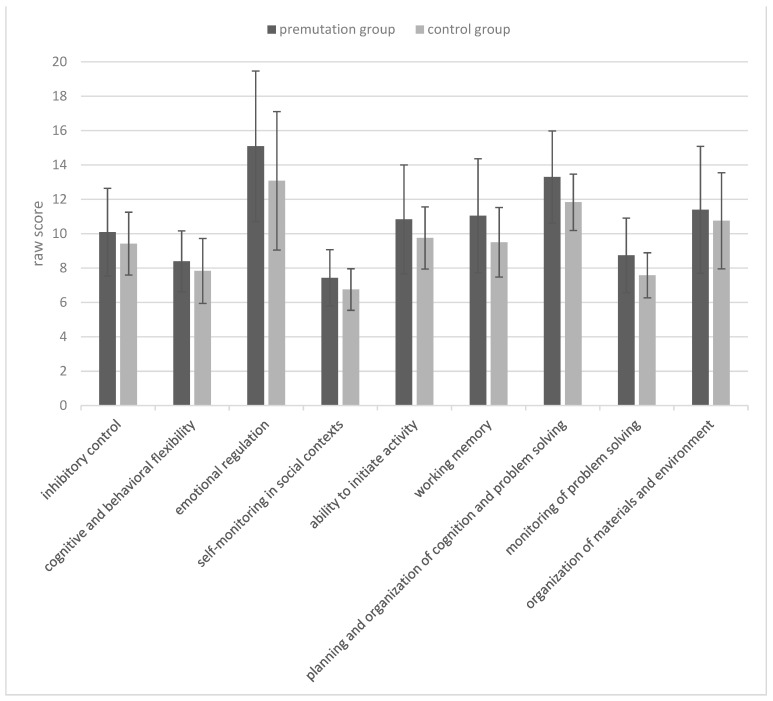
Means (in raw scores) and standard deviations for each subtest of the BRIEF-A questionnaire and for each group. Cronbach’s Alpha between the subtests was good (α = 0.88).

Note that both groups performed within the norm on all the tests. A one-way multivariate analysis of variance was run to determine the effect of the premutation of Fragile X on performances on the FriGvi subtests and digit span tests (including total raw score and standard score) as well as the phonemic and semantic fluency tests. The differences between the premutation group and the control group on the combined dependent variables were not statistically significant for the memory tests (i.e., digit span test and TMT) [F(7, 26) = 0.51, *p* = 0.81; Wilks’ Λ = 0.87; partial η^2^ = 0.12], and for the fluency tests (i.e., phonemic fluency and semantic fluency) [F(6, 28) = 1.77, *p* = 0.14; Wilks’ Λ = 0.72; partial η^2^ = 0.27]. Another one-way multivariate analysis of variance was run to determine the effect of the premutation of Fragile X on performances in BRIEF and TMT. The differences between the premutation group and the control group on the combined dependent variables was statistically significant [F(4, 30) = 40.7, *p* < 0.001; Wilks’ Λ = 0.15; partial η^2^ = 0.84]. Follow-up univariate ANOVAs showed that raw scores in the BRIEF questionnaire were significantly higher [F(1, 33) = 117.17, *p* < 0.001; partial η^2^ = 0.78] in the premutation group (M = 96.3, SD = 19.77) compared to the control group (M = 86.5, SD = 10.95), suggesting more difficulties related to executive functions. Figure 1 and Figure 2 describe the distribution of the BRIEF scores, as well as the means and standard deviations in the different subtests, respectively.

**Figure 3:**
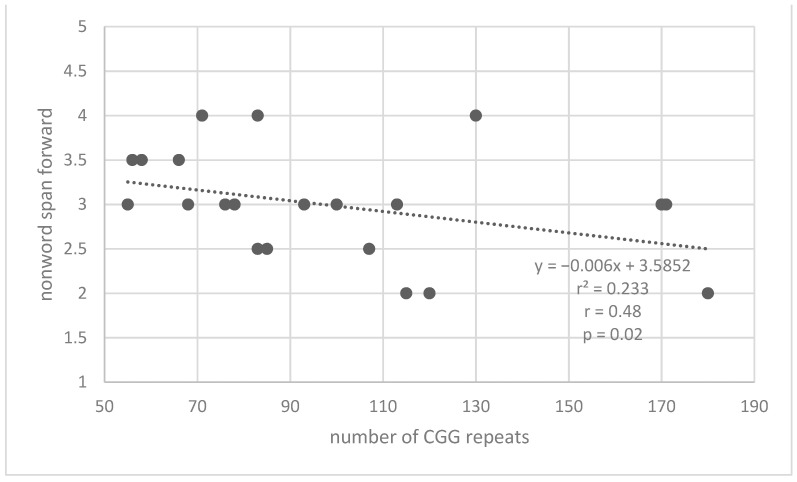
Correlation between the number of repeats on CGG in the *FMR1* gene and the nonword span (FriGvi).

**Figure 4:**
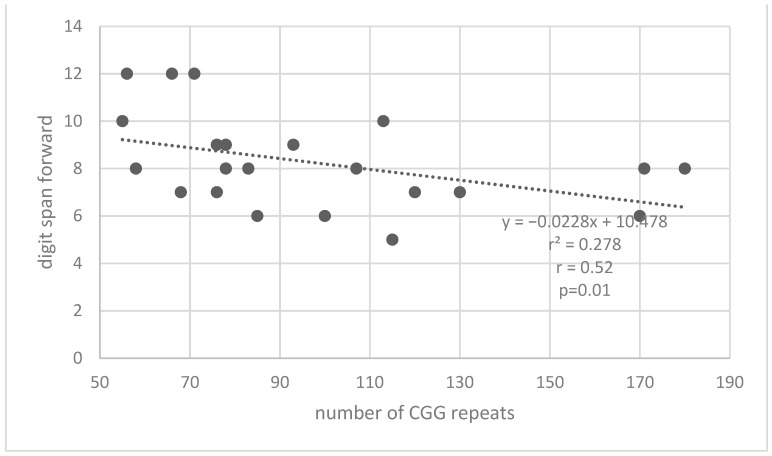
Correlation between the number of repeats on CGG in the *FMR1* gene and the digit span-forward test.

Pearson correlations were conducted between the performances on the phonological working memory tests (forward and backward digit span and nonword repetition) and the number of repeats in the premutation group to test the hypothesis regarding the relationship between cognitive functions and the number of repeats of the CGG sequence. It was found that there was a significant negative correlation between the number of repeats and the performance in the nonword span test from FriGvi [r = −0.48, *p* = 0.02] and a significant negative correlation between the number of repeats and performance in digit span forward test [r = −0.52, *p* = 0.01]. Figure 3 and Figure 4 describe the correlations between the number of repeats and the performance in the memory tests. With the increase in the number of repeats of CGG in the FMR1 gene there was a decrease in memory for nonwords and number words. No statistically significant correlations were found between the number of repeats and digit span backward test. In addition to the correlations between task performance and the number of repeats among premutation carriers, a correlation between the two tasks that are affected by the number of repeats was examined. A significant moderate positive correlation was found between the digit span forward test and nonword span test from FriGvi [r = 0.48, *p* = 0.02]. Good performance on one of the tasks also predicts good performance in the other task.

## 4. Discussion

In the present study, females with the Fragile X premutation were evaluated for executive functions and phonological working memory abilities. In addition, we examined whether there is a relationship between these abilities and the number of repeats on the nucleotide sequence CGG in the *FMR1* gene. The main finding of the present study is that female carriers perform more poorly on the executive function test (the BRIEF questionnaire) as compared to controls, but no difference was found in memory and verbal fluency tasks. An additional finding was the inverse association between the number of repeats of CGG and phonological working memory (i.e., digit span and nonword repetition) with less executive abilities linked to a higher number of CGG repeats.

We found significant differences in executive functions between female premutation carriers and the control group. Female premutation carriers reported a higher frequency of difficulties related to executive functions compared to the control group of women with normal *FMR1* alleles. Specifically, female premutation carriers experienced more difficulties in inhibition, cognitive and behavioral flexibility, emotional regulation, self-monitoring in social contexts, ability to initiate activity, working memory, planning and organization of cognition and problem solving, organization of materials and the environment, and monitoring of problems compared to the control group. Thus, female premutation carriers may have had to invest more mental resources to achieve the same level of functioning in everyday life as the control group. However, the findings have to be interpreted with caution because overall, the performance of female premutation carriers was within the norm.

The present findings are consistent with previous studies on male premutation carriers [10,29,62]. For example, an accelerated decline in executive functions, including visual working memory and inhibitory control, was found in a group of male premutation carriers compared to the control group [10]. Developing FXTAS was also found to be associated with deterioration in inhibitory control, planning and problem solving, and slowing of manual movement [10] The present findings are also consistent with some previous studies that assessed executive functions in female premutation carriers with a self-report questionnaire examining ADHD-related symptoms [51] as well as with findings on reduced processing speed, impaired response inhibition, and reduced attention and working memory [63]. In some studies, female carriers were found to have difficulties with planning and organization, which were evaluated using disfluency measures [15] and working memory measures. However, other studies did not find differences between female premutation carriers and a control group in executive functions [9], including the magnitude comparison task [27], cognitive flexibility, and short-and long-term memory tasks [51]. Possible reasons for the difference between the studies include the age of the participants [1,52], as well as the various measures used in these studies [1]. Overall, the findings of the present study support the notion that executive dysfunction might be a primary cognitive impairment among Fragile X premutation carriers in younger and older females and regardless of a FXTAS diagnosis. It is important to bear in mind that the premutation and aging have a synergistic effect on cognitive impairment among older female Fragile X premutation carriers, even in those without symptoms of FXTAS [52]. Additionally, the fact that female premutation carriers with FXTAS show more pronounced deficits in executive functions compared to female premutation carrier without FXTAS may suggest that some difficulties in executive functions could predict later signs of FXTAS [52]. However, further studies are needed to support this claim.

The variability in the number of CGG repeats in the *FMR1* gene among carriers allowed us to examine the correlation between genetic changes and cognitive performance in order to make inferences regarding the relationship between the size of the mutation and the phenotype. The examination of the association between phonological working memory abilities and the number of CGG repeats for each female tested revealed that an increased number of CGG repeats is linked to reduced scores for phonological working memory. The number of repeats could explain 48% and 52% of the variability in the performance on memory tests, repetition on nonword spans and digit spans, respectively. Both the digit span task and nonword-span task are relatively pure measures examining the capacity of phonological working memory [45]. However, the nonword span task involves no lexical knowledge. Therefore, performance on this task is based almost exclusively on immediate information stored in short-term memory [47]. Previous studies suggest that nonword span and digit span are influenced by genetic factors [38,64,65,66]. Nonword span repetition is a behavioral marker for language impairment and/or a history of language impairment in children [48,66,67]. Similar findings were shown in male premutation carriers with a negative correlation between the number of repeats and working memory [1,32]. In contrast, Moore et al. (2004) found no correlation between working memory and genetic variables in premutation male carriers [20].

Although several studies have shown a curvilinear association between the size of the *FMR1* CGG repeat and a group of symptoms in premutation carriers, correlations with other measures were also assessed. Jiraanont et al. (2017) studied whether molecular phenotypes in the *FMR1* gene, such as methylation status, number of CGG repeats, *FMR1* mRNA, and the expression levels of the *FMR1* protein could account for the clinical phenotypes that are observed in female premutation carriers including executive functions (working memory, response control, and attention) and psychiatric problems. The findings suggest no significant correlations between the molecular measures and clinical phenotypes in this sample. The authors suggested that other factors, including environment or additional genetic changes, may have an impact on the clinical phenotypes [9].

The present study shows robust findings on the association between the number of CGG repeats in the gene *FMR1* and working memory among female carriers with the premutation. The findings of this study suggest a genotype–phenotype link in females with the *FMR1* premutation. It has been reported that certain neural networks, such as those in the prefrontal cortex that serve verbal working memory, may be more susceptible to mRNA overproduction and toxicity [6]. Thus, CGG repeats may influence brain areas associated with working memory, resulting in a genotype–phenotype association.

Some researchers argue that the effect of the number of repeats on various psychological and cognitive characteristics, including executive functions, can only be seen in carriers developing FXTAS, a neurodegenerative disorder unique to premutation carriers and to carriers with more than 100 repeats of CGG [12,14]. According to Cornish et al. (2008), when there is a high number of repeats in the premutation range, there may be some deficiency in the *FMR1* protein in addition to the mRNA toxicity [12]. Moore et al. (2004) argue that deficits in executive functions and memory may be part of the premutation phenotype rather than limited to a subset of carriers with FXTAS or carriers with a high number of repeats [20]. Additional support is provided by the current study, which found that in female carriers, there is a relationship between the number of repeats and the performance of phonological working memory in the range of 55 to 180 repeats.

In healthy subjects, genetic variability between individuals may become manifest in cognitive performance and/or behavior. Nevertheless, genotype–phenotype correlations are complex for a number of reasons. Firstly, the link between a particular gene and behavior or cognitive ability is not always direct [68,69]. Secondly, genes interact with each other and with the environment, and the apparent behavior may be the result of all of these interactions [69,70]. However, the findings of the current study, particularly the relationship between the number of repeats and tasks examining phonological working memory contribute to the growing body of knowledge about the relationship between the *FMR1* gene genotype, executive functions, and phonological working memory, especially in conditions that are not considered disabilities. However, most female premutation carriers who participated in this study did not report significant difficulties in acquiring reading and writing in Hebrew or in acquiring a foreign language, which are difficulties expected among people with deficits in phonological working memory. Furthermore, quite a few of them had advanced degrees and many years of education. This attests to the impact of the environment and education, which may have helped them to compensate and overcome the difficulties posed by the genotype and to develop effective strategies [68].

The limitation of the present study is the small sample size. Further studies with larger experimental and control groups are recommended. However, even with the present relatively small sample, important differences were found in executive functions between females with the premutation and the control group. Additionally, because most mothers (15/23) had disabled children, we cannot rule out the possibility that stress and anxiety affected their performance.

Finally, children with Fragile X have mothers who are carriers of the premutation. Treatment programs for these children should also address possible difficulties in executive functions and working memory experienced by their mothers.

## Figures and Tables

**Table 1:** Summary of means and standard deviations of the executive functions questionnaire (BRIEF-A), the visual attention test (TMT), the phonologiocal working memory tests (the digit span and the FriGvi subtests), and the verbal fluency measures for the premutation group and the control group.

Measures		Premutation Group	Control Group
BRIEF-A	raw score	96.3 (19.78)	(10.95) 86.5
	standard score	47.39 (9.68)	42.5 (5.43)
TMT (seconds)	Part A	22.28 (10.7)	20.48 (7.58)
	Part B	55.419 (29.71)	41.212 (10.59)
Digit span (number of items)	forward	8.19 (1.91)	8.42 (1.5)
	backward	6.27 (2.57)	6.17 (2.16)
	total raw score	14.9 (3.84)	14.58 (3.2)
	total standard score	8.59 (2.57)	8.41 (2.15)
FriGvi (memory span)	basic word span	4.81 (0.69)	5.13 (0.64)
	phonological similarity span	3.85 (0.77)	4.04 (0.33)
	long word span	3.96 (0.37)	4.04 (0.25)
	nonword span	2.92 (0.61)	2.96 (0.33)
FriGvi (effect size)	phonological similarity effect	0.96 (9.67)	1.08 (0.66)
	length effect	0.84 (0.62)	1.08 (0.59)
	lexical effect	1.88 (0.76)	2.16 (0.49)
Phonemic fluency (number of items)	Bet (/b/)	13.65 (4.12)	14 (4.88)
	Gimel (/g/)	12.92 (4.0)	13.16 (2.2)
	Shin (/S/)	13.80 (3.74)	15.58 (5.43)
Semantic fluency (number of items)	animals	22.34 (4.67)	26.66 (3.47)
	fruits and vegetables	24.11 (4.72)	27.41 (4.23)
	vehicles	15.15 (3.69)	18.58 (5.41)

## Data Availability

Data supporting reported results can be found in the following link https://docs.google.com/spreadsheets/d/1s3aSK1bYDg7Kk3aZRm5INyxqtCVGltu_/edit?usp=share_link&ouid=101018766358984964672&rtpof=true&sd=true, (accessed on 1 March 2023).

## Supplementary Materials

The following supporting information can be downloaded at: https://docs.google.com/spreadsheets/d/1s3aSK1bYDg7Kk3aZRm5INyxqtCVGltu_/edit?usp=share_link&ouid=101018766358984964672&rtpof=true&sd=true.
